# Amalgam and How to Manipulate It

**Published:** 1895-09

**Authors:** A. C. Hewitt

**Affiliations:** Chicago, Ill.


ARTICLE V.

### AMALGAM AND HOW TO MANIPULATE IT.

DR A. C. HEWITT, CHICAGO, ILL.

The cavity should be prepared as conscientiously as for gold filling, sterilized and desiccated. To aid in doing the latter readily the following may be used as dentinal desiccant:

R.—Alcohol (pure) . . fl. ʒ v

Chloroform . . . fl. ʒ iij.

Beta naphthol . . grs. v.

M. Apply to flood the cavity thoroughly and evaporate with warm or heated water.

I use the term "desiccated" in the sense of the definition given by Dunglison (Med. Dict., 21st edition), *i. e.*, "draining, drying." Not only should salivary moisture be removed, but that lying in the tubules, the dentinal plasmasomes, and the unctuous film along broken enamel rods. I lay stress on this part of the work, for if moisture lies back of the imposed plastic, especially if the alloy contains copper, there will be oxidation and precipitation of salts to blacken tooth substance and invite influx of oral fluids.

Unless we realize how difficult it is to bar moisture from any place, we will underestimate the need of the care advised. Next, the surfaces to be covered in by the amalgam should be coated well with some resinous solution for two purposes. First, to bar moisture, and second to form a stick base in and on which to grind particles of the alloy. For this purpose the following is what I have used with gratifying success:

R.—Sandarach varnish,
Damar varnish . . aa fl. ℥j.
Alcohol (absolute) . . fl. ℥j.
Beta naphthol . . . gr. v.

M. Apply as varnish.

When the liquids have evaporated, leaving the resins as a lining for the cavity, the dentine and enamel should receive another coating, this time of amalgam burnished on to the varnish with a smooth-point amalgam plugger flat-faced or "shot" pointed, till the cavity floor and walls, take on a mirror-like surface. By thus burnishing the amalgam on the walls, every tubule and enamel interstice will be filled and brightened over.

The amalgam for this first coating may be well saturated with mercury, but only sufficient of the moist mass left to form a thin coating, all surplus possible being "poked" or burnished out of the cavity. From thence on, amalgam wrung through heavy muslin or chamois only is to be used; one layer after another burnished on the preceding one, each added mass being as dry as can be and cohering to the preceding one. If free mercury is thus brought to the surface of the plug, it should be "poked" or burnished out before another dry mass is added. Keep the growing mass dry. Thus build till the cavity is "rounding full," care having been taken that every undercut, fissure, nook and corner is densely filled, not with free mercury, but with the alloy. When the cavity is more than full, "rounded over" like a bushel of potatoes in a basket, coax the surplus mercury to the highest point by gentle pats or tapping with the plugger, and while thus atop, absorb it (free mercury) with tin foil No. 4, rolled into balls, or what is better, "bricks" of Watt's crystal gold No. 2, freshly annealed in a lamp flame. When the free mercury is thus disposed of the surplus amalgam to be burnished down to a proper level with a hand-shot point, or a steel "Herbst" point in an engine hand piece, rapidly driven, moving the instrument from the center toward and over the enamel borders. Thus the line of free mercury, always tending to cling to enamel border, will be taken up and carried off by the dryer surplus portion of the filling. Before removing the dam, varnish the plug. In mixing the amalgam, the bare pulm of the hand and the bare finger should never be used.

Enough oil and moisture, from a bare palm, can be worked out to saturate an ordinary sized mass for a filling. Dry the cavity; keep it dry. Prepare the amalgam free from oils and water; pack densely; absorb surplus mercury. At a future sitting, polish the filling. If good alloy is used and this care is taken in the manipulating, but few workers will live long enough to see the need of a repair.

Of course, some cavities can only be filled "submarine." Then try gutta-percha till above high-water mark.—*Items of Interest.*

---

